# Supplementary material for: Are carbon emissions trading and green financial instruments synergistic? -Comprehensive quantitative research based on content analysis
Source: PLoS One. 2024 Mar 7;19(3):e0298601. doi: 10.1371/journal.pone.0298601 (PMC10919601; doi:10.1371/journal.pone.0298601)
Supplement: S1 Data — (ZIP) [file pone.0298601.s001.zip › Data/地级市Z_F工作报告.zip!/║╙─╧/╫ñ┬φ╡Ω╩╨/└┤╫╘╣1⁄2╓┌║┼í╛┬φ┐╦╩2╛▌═°í┐_2012.docx]

各位代表:

现在，我代表市人民政府，向大会作工作报告，请予审议，并请各位政协委员提出意见。

折叠编辑本段过去五年工作的回顾

2007年以来，市二届人民政府在省委、省政府和市委的正确领导下，在市人大、市政协的监督支持下，以邓小平理论和"三个代表"重要思想为指导，深入贯彻落实科学发展观，坚持"四个重在"实践要领，抢抓中原经济区建设战略机遇，多策并举，开拓创新，经济社会发展取得了显著成就，较好地完成了市二届人大各次会议确定的目标任务。

折叠五年来，综合经济实力显著增强

认真落实中央和省各项决策部署，采取有效措施，积极应对复杂形势，保持了经济平稳较快发展。去年，全市生产总值达到1259.9亿元，较2006年翻了一番，年均增长12%。地方财政一般预算收入完成47.1亿元，是2006年的2.9倍，年均增长23.8%;税收收入占一般预算收入的比重达到74.4%。社会消费品零售总额447.23亿元，是2006年的2.39倍，年均增长18.7%。粮食产量达到136.97亿斤，连续4年稳定在130亿斤以上。项目建设扎实推进，共实施重点项目630个，累计完成投资548.5亿元。华润古城2×30万千瓦净化燃烧机组、国电驻马店热电2×33万千瓦供热机组、白云纸业20.4万吨文化用纸、顺达化工20万吨醋酸等一批重大项目建成投产。规模以上工业企业1382家，较2006年增加817家。全社会固定资产投资完成747.2亿元，是2006年的3.5倍，年均增长28.1%。

折叠五年来，经济结构持续优化

二、三产业比重达到73%，比2006年提高5.3个百分点。现代农业快速发展，一批特色农产品生产规模扩大，各类产业化组织发展到2085家，市级以上龙头企业达到316家，比2006年增加243家，去年完成农业增加值339.9亿元，年均增长4.6%。工业发展质量效益提升，食品、能源和煤化工、建材、装备制造、轻纺、医药等支柱产业不断壮大，骨干企业的支撑作用明显增强。华顺阳光、施科特光电、华润风电等一批新兴产业项目顺利实施。规模以上工业增加值、利润分别达到370.3亿元和119.2亿元，年均分别增长22.7%和46.6%。12个产业集聚区入住企业1034家，产业集聚度明显提升。服务业稳步发展，金融机构各项存款余额1173.7亿元，各项贷款余额540.81亿元;商品房销售额达123.9亿元，旅游综合收入达53.5亿元，分别是2006年的21.6倍和7倍，全市第三产业增加值达到375亿元，年均增长14.2%。自主创新能力逐步提升，创建国家、省级企业技术中心19家，省、市级工程技术研究中心63家。节能减排和环保工作取得新进展。

折叠五年来，城乡面貌明显改观

完成了中心城区总体规划大纲和165个乡镇建设规划编制工作，全市城镇化率由20.6%提高到31.54%。中心城区累计投入103亿元，新建、改造了一批游园广场、环卫、供排水、市政道路等基础设施，城市供水、供气、供暖能力提高一倍以上，污水、垃圾无害化处理率达到80%，绿地率、绿化覆盖率分别达到34%、40.2%，成功创建了中国优秀旅游城市、国家园林城市和省级卫生城市，被评为"省文明城市创建工作先进市"。县城、小城镇和新型农村社区试点建设稳步推进，文明乡镇创建和清洁家园活动成效明显，城镇吸纳聚集能力进一步增强。农村生产生活条件持续改善，完成了一批水库除险加固、河道治理、灌区改造等工程建设，新增有效灌溉面积245万亩，旱保田185万亩，新增农村沼气25.8万户，解决了153万农村人口安全饮水问题，50.5万农村人口实现脱贫。高速公路、干线公路和"村村通"工程建设成效明显，四级以上公路通车总里程达1.25万公里。

折叠五年来，改革开放深入推进

国有企业改革不断深化，12家国企完成改制任务;行政审批制度改革扎实推进，共取消、下放审批事项23项;金融改革成效明显，组建了一批政府融资平台和投资担保公司，驻马店商业银行更名为驻马店银行，农信社改革顺利推进;基层医疗卫生机构综合改革圆满完成，全面实施了国家基本药物制度，财税、文化、教育、水利、集体林权等领域改革取得新的成效。积极创新土地、金融和劳动力等要素保障机制，累计报批建设用地11万亩，新增贷款余额373亿元，职业技能培训74.8万人，有效缓解了发展瓶颈制约。以举办全国"农洽会"为载体，大力开展招商引资，积极承接产业转移，推进外向型经济发展，累计引进市外项目1900多个，实际到位资金509亿元;新批外商投资企业93家，实际利用外资5.6亿美元;完成进出口总额8.98亿美元，其中出口7.06亿美元。

折叠五年来，人民生活水平明显提高

城镇居民人均可支配收入、农民人均纯收入分别达到15795元、5804元，年均增长14.1%和14.8%。坚持把更多的财力向民生倾斜，每年承诺的实事得到较好落实，2011年民生支出153.5亿元，是2006年的4.1倍。全面实现城乡免费义务教育，生均教育经费持续提高。积极推进中小学布局调整，新建、改造了一批中小学校。大力实施职教攻坚计划，在校生达到10万人。加强文化服务体系建设，市图书馆、职工文化活动中心建成投用，市博物馆、群艺馆、广电中心建设进展顺利。新建改造了一批县级人民医院、县中医院和乡镇卫生院、城市社区卫生服务中心，开工建设了市中心医院病房楼、精神病院综合楼等一批重点项目。城镇居民医疗保险参保人数达74万人，新农合参合率达98.2%，城乡医疗卫生保障水平明显提升。人口自然增长率控制在6‰以内，稳定了低生育水平。群众健身运动蓬勃开展，竞技体育水平不断提高。切实加强就业再就业服务，累计城镇新增就业38万人，下岗人员再就业13.2万人，其中"40"、"50"人员5.5万人，城镇登记失业率为3.67%。社会保障体系不断完善，新农保试点顺利开展，企业退休人员养老金标准逐步提高，城乡低保实现应保尽保，养老、工伤、失业和生育等参保范围逐年扩大。保障性住房建设加快推进，累计建成保障性住房3.49万套。信息化步伐明显加快，移动通信基站达到4125个，手机用户达到300万户。

折叠五年来，和谐社会建设取得新进展

切实加强公民道德建设，扎实开展各类群众性精神文明创建活动，公民道德水平和城乡文明程度不断提升。自觉接受人大法律监督和政协民主监督，人大代表议案和政协委员提案办结率达100%。深入开展普法教育，"五五"普法教育全面完成，"六五"普法工作扎实推进。持续推进"两转两提"，切实加强反腐倡廉建设，积极开展政风行风评议活动，政府自身建设得到加强。加强和创新社会管理，完善群众工作体系，认真做好信访稳定工作，积极化解各类矛盾纠纷，依法打击各种违法犯罪活动，人民群众的安全感明显提升。全面落实安全生产责任制，切实加强对重点领域的专项整治，有效防止了重特大事故发生，保持了社会大局和谐稳定。高度重视驻驻部队、国防后备力量和人民防空建设，认真做好拥军优属、拥政爱民和军民共建工作，再次荣获全国"双拥模范城"称号。圆满完成了第六次人口普查和对口支援四川江油灾后重建任务。人事、科技、审计、统计、气象、民族宗教、外事侨务、红十字会、史志、档案、残疾人等工作都取得了新的成绩。

各位代表!过去的五年，是经济平稳较快发展，综合实力显著增强的五年;是经济结构持续优化，城乡面貌明显改观的五年;是人民生活水平大幅提高，社会大局和谐稳定的五年。特别是去年以来，我们借助中原经济区建设的东风，以项目建设为着力点，确定"经济发展十项重点工作、四项保障机制"，坚持项目建设周例会和领导分包重点项目制度，强化各项工作机制，全市呈现大上项目、上大项目、赶超发展的良好局面，有力地推动了经济社会的快速发展。五年的实践充分证明，推动驻马店的发展，办好驻马店的事情，必须始终坚持把发展作为第一要务，必须始终坚持以项目建设为抓手，必须始终坚持改革开放不动摇，必须始终坚持保障和改善民生。回顾五年的奋斗历程，我们深切体会到，驻马店所取得的这些成就来之不易，是省委、省政府和市委正确领导的结果，是全市上下立足改革、着眼建设、齐心协力、努力奋斗的结果。在此，我代表市人民政府，向工作在全市各条战线的广大工人、农民、知识分子、干部、驻驻部队指战员、武警官兵、公安民警，向所有关心支持政府工作的各位人大代表、政协委员、离退休干部和民主党派、工商联、无党派人士，表示崇高的敬意和衷心的感谢!

各位代表!过去的五年，尽管我市经济社会发展取得显著成绩，但也应清醒地看到，人口多、基础差、底子薄的基本市情没有根本改变，农业大市、工业小市、财政穷市的基本状况没有根本改变，发展中仍面临不少困难和问题，主要体现在:经济总量小，人均水平低，综合实力不强，区域竞争压力较大;经济结构层次低，发展方式粗放，工业支撑作用不强，三产发展相对滞后，结构调整任务艰巨;项目建设层次不高，产业集聚区发展缓慢，土地、资金、人才等要素瓶颈制约加剧，经济发展后劲不足;城镇化率低，农业产业化水平不高，社会事业欠账较多，统筹城乡协调发展压力较大;发展环境不够宽松，政府行政效能、服务质量有待提高，公务员队伍建设有待加强等。对此，我们务必保持清醒忧患，采取有效措施，认真加以解决，既要争取多方支持，又要增强发展的内生动力，自我加压，赶超发展，决不辜负全市人民的期望和重托。

折叠编辑本段今后五年的总体要求和目标任务

纵观发展形势，把握阶段特征，当前驻马店已经站在新的历史起点上，步入了加快发展的快车道，正处于乘势而上、加快崛起的关键时期。今后五年，我们既面临着国家推进中原经济区建设、继续加大三农投入、加强社会保障，以及沿海产业加速转移等难得机遇，也面临着结构性矛盾突出，资源环境约束加剧，区域竞争压力增大等新的挑战。面对新的机遇和挑战，我们必须时刻保持清醒的头脑，进一步增强发展的紧迫感和责任感，敏锐把握时代脉搏，深刻认识市情，找准发展定位，凝聚各方力量，努力把我市的现代化建设事业不断推向前进。

今后五年政府工作的总体要求是:以邓小平理论和"三个代表"重要思想为指导，深入贯彻科学发展观，认真落实省第九次党代会和市第三次党代会精神，坚持"四个重在"实践要领，按照"三个一"的工作思路，立足中原经济区建设大局，围绕"一城两区四基地"战略定位，以推进"经济发展十项重点工作"为载体，把稳增长、调结构、控物价、惠民生、促改革、保稳定有机结合起来，突出抓好项目建设、招商引资、城乡统筹、民生改善，持续探索"三化"协调科学发展的路子，为实现驻马店富民强市、加快崛起而努力奋斗。

今后五年政府工作的主要奋斗目标是:

--经济发展再上新台阶。全市人均生产总值、地方公共财政预算收入实现"两个翻番"。

--经济结构进一步优化。二、三产业占生产总值的比重达80%以上，规模以上工业增加值占全部工业增加值的比重达80%以上。力争建成3个以上主营业务收入超百亿元的企业集团，6个以上超百亿元的特色产业集群，4个以上超500亿元的支柱产业。

--城镇化步伐明显加快。建设覆盖全市城乡的新型城镇体系。中心城区面积接近100平方公里，人口接近100万人。县城人口力争达20万以上。城镇化率达40%以上。

--农业现代化水平加快提升。粮食总产量稳步提升，综合生产能力稳定在140亿斤以上;培育壮大一批国家级农业产业化龙头企业，引进和培育一批知名农产品加工品牌，建成在全国有重要影响的农产品加工产业集群，打造成豫南最大的农产品贸易中心。

--改革开放继续深化。重要领域和关键环节的改革取得重大进展，经济发展的动力明显增强;开放型经济持续发展，进出口总额、实际利用外资年均分别增长20%。

--人民生活水平进一步提高。城乡居民收入明显增加，群众生活水平显著提高;生态环境进一步改善;社会保障体系进一步健全，社会事业全面进步，社会大局和谐稳定。

实现上述目标，我们必须抓住中原经济区建设的机遇，突出"一个主题"，把握"一条主线"，围绕"中心任务"，谋发展，求突破。突出"一个主题"，就是以科学发展为主题，按照"一城两区四基地"战略定位，积极探索不以牺牲农业和粮食、生态和环境为代价的以新型城镇化为引领的"三化"协调科学发展的路子。把握"一条主线"，就是以加快转变经济发展方式为主线，调结构、促转型，推动经济结构优化升级，在发展中促转变、在转变中谋发展。围绕"中心任务"，就是以富民强市、加快崛起为中心任务，坚持富民优先、强市为要，努力实现驻马店加快崛起的目标。在工作指导上，着力把握好以下几点:一是努力扩大投资规模。继续以项目建设为抓手，认真谋划筛选项目，招商引资大上项目，积极争取上级支持，全力抓好项目实施，努力扩大投资规模，拉动经济快速增长。二是积极调整经济结构。突出抓好产业结构和城乡结构调整，培育壮大支柱产业，提高产业集聚区发展水平，着力解决产业竞争力弱、城乡差距大等问题，促进经济社会又好又快发展。三是继续深化改革开放。坚持以改革创新破解发展难题，以扩大开放突破瓶颈制约，努力消除体制机制障碍，不断增强经济社会发展动力和活力。四是着力保障改善民生。坚持发展依靠人民，发展为了人民，推动社会事业全面发展，扩大社会保障覆盖面，加强社会管理创新，提高群众生活水平，提升公共服务质量，让人民群众真正成为改革发展的受益者。五是切实加强政府自身建设。坚持以领导方式转变加快发展方式转变，认真落实"三具两基一抓手"，深入推进"两转两提"，以优良的工作作风和良好的精神状态，带领全市人民艰苦奋斗，开拓创新，真抓实干，努力谱写驻马店加快崛起的新篇章。

折叠编辑本段2012年工作的主要目标和任务

2012年是新一届政府工作的开局之年，也是实施"十二五"规划的关键之年。我们一定要按照"持续求进"的总基调和"三个一"的工作思路，采取针对性措施，全力做好各项工作，为今后五年的发展开好局、起好步。今年经济社会发展主要目标是:生产总值增长10%以上，规模以上工业增长18%以上，地方公共财政预算收入增长13%以上，全社会固定资产投资增长21%以上，社会消费品零售总额增长17%，居民消费价格涨幅控制在4%左右，人口自然增长率控制在6.5‰以内，新增城镇就业人员5万人，城乡居民收入较快增长，节能降耗完成省定任务。

今年以来，由于受欧债危机和国内宏观调控政策的影响，经济社会发展面临更加复杂严峻的形势，经济下行压力加大，固定资产投资增长乏力，财政收支矛盾加剧，要完成确定的各项目标，任务非常艰巨。我们必须坚持把投资作为主要举措，大力推进项目建设，千方百计扩大投资规模，培育新的经济增长点。重点抓好以下八个方面的工作:

(一)统筹城乡建设和发展，强化新型城镇化引领作用。坚持以新型城镇化引领，既是推动"三化"协调科学发展的治本之策，也是应对复杂局面、扩大内需、拉动投资的现实选择，必须把加快新型城镇化作为战略性举措，统筹推进中心城区、县城、小城镇和新型农村社区建设，加快构建符合驻马店实际的现代城镇体系。着力推进中心城区发展。以打造地方特色突出、城市标志形象明显的山水园林、生态宜居城市为目标，按照"一中心五组团"空间布局，加快中心城市总体规划和新区规划修编工作，继续抓好专项规划和控制性、修建性详细规划编制，提高控规覆盖率。启动金顶山和宿鸭湖组团建设，推动中心城区与外围组团互动发展。加快新城区配套建设和老城区升级改造，大力实施市政道路、水系治理、园林绿化、环卫设施建设工程，积极推进薄山水库引水工程、第二污水处理厂以及火车站广场升级改造、石武高铁客运站场等重点项目建设，切实加快城中村、旧城区和棚户区改造步伐。健全城市管理长效机制，深入推进城市精细化管理，不断巩固中心城区"三项整治"成果，进一步改善城市面貌。加快县城和重点镇建设。认真实施城乡建设三年大提升计划，全面推进供排水、供电、供气、生态等基础设施建设，培育主导产业，强化产业支撑，提升县城综合服务功能和产业集聚能力。因地制宜发展小城镇，重点抓好具有资源、农产品加工和文化旅游特色的小城镇建设，完善城镇功能，加强环境整治，提升发展层次。加快城镇户籍制度改革，解决好进城务工人员的安居、子女就学、社会保障等问题，促进农村人口有序转移。积极稳妥推进新型农村社区建设。全面完成新型农村社区规划编制工作，以城市新区、产业集聚区、乡镇和旅游景区周边区域为重点，因地制宜，量力而行，建设一批标准高、规模大、功能全的新型农村社区，各县区要开工建设2个以上规划入住人口不少于1万人的新型农村社区。积极破解土地、资金两大难题，推动土地集约利用、农业规模经营、农民多元就业，确保农民进得来、稳得住、能发展、可致富。

(二)加快推进产业结构调整，提升新型工业化主导作用。按照产业发展规划，切实加大政策、资金和技术的支持力度，促进产业总量扩张、结构优化、水平提升。强化工业支撑。积极引导要素资源向优势产业、骨干企业集中，加快一批龙头型、基地型项目建设，带动配套产业发展，力争食品行业主营业务收入达到460亿元以上，轻纺、能源煤化工行业均达300亿元以上，建材行业达250亿元以上，装备制造业达220亿元，医药行业达80亿元。深入推进骨干企业战略重组和链条项目建设，抓好"双50"企业培育，强化上下游企业合作，促进链条延伸和升级改造，发展壮大一批大型企业和企业集团，力争主营业务收入超80亿元和超50亿元的企业各1家、超40亿元企业2家、超10亿元企业15家。加快生物医药、新能源、电子设备等新兴产业的培育，积极推进风电、秸秆发电、光伏太阳能、通用天方新药产业化等项目的实施和前期工作。加强运行监测，继续强化对企业的服务，建立健全政策导向、市场开拓、融资服务、困难企业帮扶等长效机制，主动帮助企业解决实际困难，切实保障要素供应，确保工业经济健康运行。发展壮大现代服务业。全面提升商贸流通、餐饮娱乐等生活性服务业，积极推动金融保险、社区服务等高成长性服务业发展，突出抓好豫南农产品交易中心、恒兴仓储物流配送、市公共保税中心等重点项目建设。加快旅游资源整合，推进嵖岈山旅游集聚区、老乐山文化生态产业园建设，着力打造精品景区和精品线路。充分挖掘整合文化资源，积极培育文化企业、文化市场，做大做强文化产业。加快产业集聚区发展。按照"四集一转"和"做大做强、集中集聚、提速提升"的要求，科学调整规划布局，完善支持政策，创新管理机制，推进产城互动。加快推进集聚区外连通道、污水处理、供水供电以及职工培训、物流仓储、质量检测等公共服务设施建设，全面提升综合配套能力。综合运用项目准入、要素配置、利益分成等手段，推动关联项目集中布局，加快培育一批收入超百亿元的特色产业集群。加强自主创新能力建设。注重科技创新服务平台建设，支持有条件的企业建设国家级和省级工程技术研究中心，支持通用天方、华中正大等自主创新企业发展。大力实施质量立市战略，推进标准化体系建设，选择一批重点企业、重点产品，在政策、资金、技术上倾斜扶持，提高产品质量安全水平，扩大品牌影响力和市场占有率。加强生态环境保护。认真落实节能降耗综合工作方案，完善评价考核和奖惩机制，突出抓好高耗能行业和重点领域的节能降耗。加强污染减排工作，实施主要污染物总量预算指标管理，强化环境影响评价管理，促进环境资源优化配置。加强生态环境保护，抓好西部山区"三乱二超"综合整治，继续开展重点流域、重点区域、重点行业环境治理，抓好污水、垃圾处理设施建设和运营。

(三)持续做好"三农"工作，夯实新型农业现代化基础。围绕现代农业示范区建设，加快规划编制，抓好分步实施，逐步将我市建成全国有影响的农产品生产基地、加工基地、贸易基地。认真落实强农惠农政策。加大财政用于"三农"的支出，增加粮食生产直接补贴，认真落实重要粮食品种最低收购价和增加粮油生产大县、生猪调出大县奖励资金政策。积极争取国家、省财政资金支持，整合各类支农资金，统筹用于现代农业示范区建设。大力推进高标准粮田建设。以县为实施主体，集中投入，整体开发，统筹推进水、电、路、林等田间生产设施建设，着力打造56个万亩方、105个千亩方、70个百亩方。加快中低产田改造、大中型灌区续建配套、节水灌溉、平原洼地治理等工程建设，力争新增、改善有效灌溉面积87.6万亩，新增旱保田61.6万亩，确保全市粮食总产达到137亿斤。积极推进农业结构调整。扩大油料、食用菌、蔬菜、花木等经济作物种植面积，积极推进茶产业发展，确保油料、蔬菜种植面积分别达到400万亩、150万亩，发展良种茶园1.03万亩。加快标准化规模养殖和夏南牛产业发展，新改扩建200家标准化畜禽养殖场区。抓好林果业生产和生态林建设，完成成片造林18万亩，力争40%的农业乡镇达到林业生态乡镇标准。大力发展农民专业合作社，深入推进农业产业化经营，突出基地化、标准化、规模化，努力打造一批农业产业化集群，带动农业结构调整，促进农民增收。继续改善农村生产生活条件。加强农村安全饮水、道路、供电、通信、沼气等基础设施建设，深入推进农村环境综合整治，大力提升农业机械化水平，力争再解决44万人的安全饮水问题，新增农村沼气用户1万户，完成80个贫困村的整村推进任务，解决脱贫人口13万人。全面推进农村各项改革。全面开展农村集体土地确权登记发证，推动集体建设用地使用权有序流转。鼓励和支持农村种植大户、经济能人适度规模经营土地。深化农村金融体制创新，推进林权、农房抵押贷款试点，继续推广农信社农户联保贷款。建立健全乡镇农技推广、动植物疫病防控等公共服务机构。

(四)强力推进项目建设，着力扩大市场需求。大力推进重点项目建设，确保市、县区两级重点项目投资超过350亿元，带动全社会固定资产投资达到820亿元，努力实现"两个翻番、一个接近"的目标。工业方面，突出抓好天方药业抗生素原料药、昊华骏化合成氨原料路线改造、电动汽车科技园、施科特光电产业园、乡巴佬食品工业园、圣光鼎瑞模塑科技园、恒都食品加工等项目建设。水利方面，加快推进洪汝河治理及老王坡、杨庄、蛟停湖三大滞洪区建设，开工建设一批病险水库除险加固和7条中小河流治理工程。继续实施薄山、板桥等灌区节水改造与续建配套项目。交通方面，抓好京港澳高速漯驻、驻信段改造，京港澳高速至正阳、上蔡县城连接线等项目建设。能源方面，抓好泌阳中天风电、华润安里煤矿、吴桂桥煤矿技改、驻信成品油管道工程等项目建设。社会事业方面，抓好市职业教育园区、市中心医院新区分院、市体育中心等项目建设。对于这些项目，要按照"项目建设推进年"活动的部署，认真落实市、县领导分包重点项目以及重大项目"绿色通道"和联审联批制度，坚持项目建设周例会制度，及时协调解决项目建设中遇到的突出问题，确保重点项目建设顺利实施。着力扩大消费需求。全面落实国家调整收入分配关系的各项政策，努力增加城乡居民特别是低收入群众收入，增强居民消费能力;完善服务设施，拓展和开发消费领域，促进文化、旅游、养老、家政等服务消费;强化监管和服务，坚决打击商业欺诈、制假售假行为，让广大群众放心消费、安全消费。积极扩大出口。通过承接产业转移，引进一批发展前景好、市场潜力大的出口企业，培育新的出口增长点;优化出口商品结构，扩大优势农产品和高新技术产品出口规模，积极开拓新兴市场。

(五)加大招商引资力度，努力扩大对外开放。大力实施开放带动主战略，提升水平、拓展领域、完善机制，以开放促发展、促改革、促创新。提升招商引资水平。立足我市资源和产业优势，以现有骨干企业和产业集聚区为依托，积极吸引沿海地区食品、纺织服装、鞋业、中小型电子等劳动密集型产业向我市转移。创新招商方式，突出"招大引强"，瞄准国内外500强等知名企业，重点围绕引进龙头企业、重大项目以及产业链的关键环节，开展定向招商，力争引进超百亿元产业项目。充分利用福建河南商会、广东商会等招商合作团体，组织实施好"百亿闽资汇天中"活动，确保取得实效。以办好全国"农洽会"为载体，认真组织开展各类招商活动，招引和实施一批高质量的合作项目。加强与境外企业和投资机构的交流与合作，积极引进更多的资金、技术和人才，提高利用外资水平。拓宽开放领域。加快服务业对外开放，吸引境内外投资者进入现代物流、金融、旅游、文化等领域。深化农业开放合作，重点引进食品加工、畜牧养殖与精深加工、花卉苗木等基地型项目。扩大其他领域的对外开放，鼓励各类资本参与基础设施建设和教育、卫生、养老事业发展。完善工作机制。牢固树立开放"一盘棋"思想，建立党政机关、企业事业、社会各界广泛参与、协作联动的开放长效机制。加强对已签约项目的跟踪落实，切实提高项目履约率、资金到位率和开工投产率。强化招商引资责任，健全考评机制，完善奖惩办法，确保招商引资工作实现新突破。

(六)推进体制机制改革创新，增强发展动力和活力。全面深化各项改革，加快商贸、建筑、交通、文化等非工企业"脱钩改制"，积极推进机械、化工、食品、医药等重点行业重组，培育壮大一批竞争力强的企业集团;深化文化事业单位改革，完善文化市场综合执法体制，抓好市影剧院、豫剧团转企改制工作;继续深化行政审批制度改革，清理精简行政审批事项，推行政府绩效管理。认真落实国家财税、金融、价格等改革措施，扎实推进事业单位分类改革和医药卫生体制改革。同时，积极推进要素保障机制创新。创新投融资机制。完善金融服务体系，壮大金融产业，积极引进国内金融保险机构，扩大保险范围，规范担保行为，优化金融生态环境，提高金融业对经济发展的支撑能力。继续组织多种形式的银企洽谈会、重点项目推介会，开展好"小巨人"企业信贷培育工作，促进银企对接，努力破解中小企业融资难问题。通过整合资金、资源、资产，做大做强市县两级政府投融资平台，提高信用级别，增强融资能力。抓好重点上市后备企业培育工作。创新土地开发利用管理机制。实施差别化土地政策，计划指标重点保障市中心城区的重点项目和重要基础设施用地，建设用地增减挂钩指标和"三项整治"节余指标集中用于县域城镇及产业集聚区。大力开展治理囤地圈地、违规用地、土地闲置浪费问题专项治理，盘活存量土地资源。扎实推进农村土地整治，保障新型农村社区及非农产业发展用地。严格执行各类工程项目建设用地标准，将新增建设用地计划分配与各地节约集约用地成效挂钩。创新人力资源开发机制。加快实施职教攻坚和全民技能振兴工程，利用"阳光工程"、"人才战略工程"、"贫困地区劳动力转移培训"等平台，做好农村劳动力技能培训，确保全年完成各类培训10万人。鼓励支持专业大户、科技人员、大中专毕业生围绕现代农业发展创业，带动农民致富。

(七)努力保障和改善民生，保持社会和谐稳定。坚持为群众办好事、办实事，切实加大民生投入，让人民群众共享改革发展成果。加强就业和社会保障工作。继续实施积极的就业政策，鼓励以创业带动就业。强化就业服务，重点抓好高校毕业生、城镇就业困难群体、退役军人和残疾人就业帮扶工作，确保城镇新增就业5万人以上，新增农村劳动力转移就业7万人。继续增加企业退休人员基本养老金，提高城乡低保补助水平及优抚对象、困难群众保障标准。基本实现新农保和城镇居民社会养老保险制度全覆盖。认真解决失地农民生活保障问题，实现被征地农民社会保障全覆盖。加快实施保障性住房建设，开工建设各类保障性住房2.6万套。大力发展各项社会事业。完善农村义务教育经费保障机制，加强义务教育学校标准化建设，推进义务教育均衡发展。结合城市新区建设和旧城改造，加快实施中小学校、幼儿园新扩改建工程。落实好学前教育三年行动计划，逐步解决入园难问题。推进职教园区建设，加快汝南幼儿师范申办高等专科学校步伐。完善基层医疗卫生机构综合改革，将基本药物制度实施范围扩大到村卫生室，继续提高城镇居民医保和新农合政府补助标准和农民受益水平。争取国家和省项目支持，加大对人口大县、县级医院、乡镇卫生院的投入力度。健全公共卫生、疾病防治体系，提高应急处置能力。加强残疾人就业培训工作，落实好0~6岁残疾儿童抢救性康复任务。大力实施文化惠民工程，加快博物馆、群艺馆、广电大厦、报业大厦等项目建设，推进公益性文化设施免费开放。深入实施全民健身计划，广泛开展群众体育活动。严格落实计划生育政策，稳定低生育水平，促进人口长期均衡发展。继续做好人事、审计、统计、外事侨务、红十字会、档案、史志、地震、气象、无线电管理等工作。提高全民文明素质。树立和践行社会主义荣辱观，深入推进社会公德、职业道德、家庭美德、个人品德建设，加强青少年思想道德建设。大力弘扬艰苦奋斗、求真务实精神和新时期"三平"精神，深化拓展道德实践活动和群众性精神文明创建活动，着力塑造高尚人格，培育和谐人际关系，形成良好社会风尚。加强和创新社会管理。健全党委领导、政府负责、社会协同、公众参与的社会管理格局，全面提高社会管理科学化水平。大力推进政务诚信、商务诚信、社会诚信和司法公信建设。增强城乡社区自治和服务功能，完善基层社会管理和服务体系。深入实施"六五"法制宣传教育和依法治理规划。扎实推进"安全驻马店"创建工作，认真落实安全生产责任制，加强重点领域隐患排查整治，健全安全生产投入保障机制，坚决防止重特大事故发生。加强食品药品安全监管。加强和改进信访工作，落实重点项目风险评估机制，妥善处理土地征收、拆迁安置、涉法涉诉等方面的矛盾，保障群众合法权益。强化源头治理，积极预防、妥善处置群体性事件和突发公共事件。强化社会治安综合治理，严密防范、依法打击各种违法犯罪活动，保障人民群众生命财产安全。全面贯彻党的民族宗教政策，切实做好民族宗教工作。加强国防教育和国防后备力量建设，支持驻驻解放军、武警部队、预备役部队、民兵和人民防空建设，促进军民融合式发展。深入开展双拥共建，巩固发展军政军民团结。

(八)切实加强政府自身建设，努力提高施政水平。坚持用领导方式转变加快发展方式转变，认真落实"三具两基一抓手"，深入推进"两转两提"，努力建设为民、务实、廉洁、高效政府。坚持依法行政。严格执行人大及其常委会的决议决定，自觉接受人大法律监督和政协民主监督，认真办理人大代表建议和政协提案。强化监察、审计等专门监督，依法推进政务公开、信息公开，主动接受各方面监督。支持工会、共青团、妇联等人民团体发挥桥梁纽带作用。规范行政执法行为，强化执法责任和过错追究，提高行政执法水平。完善重大事项集体决策、专家咨询、社会公示与听证等制度，增强工作的前瞻性、系统性。提高服务水平。围绕服务经济社会发展、服务人民群众，扎实开展"转变作风、优化环境"集中整治活动，加大问责力度，重点整治行政不作为、乱作为等行为。健全审批运行机制，减少审批环节，压缩审批时限，推动政府工作高效运转。积极开展争做"人民满意的公务员"活动，努力建设一支政治坚定、业务精湛、作风过硬的公务员队伍。强化督查落实。创新方式方法，注重科学运作，精简会议和文件，减少迎来送往，严格控制和规范各种检查、评比、达标活动，提高办事效率。完善督查督办机制，对市里的重大决策和重点工作，坚持做到每月督查、每季度通报、每半年汇总、年底总结考评，确保各项任务的落实。强化政府绩效管理，健全绩效考核和激励约束评价机制，对埋头苦干、成绩突出的表彰奖励，对失职渎职的严肃追究责任。加强廉政建设。大力倡导勤俭节约、艰苦朴素之风，坚决反对讲排场、比阔气、铺张浪费等行为。认真落实党风廉政建设责任制，扎实推进惩治和预防腐败体系建设，不断拓展从源头上预防腐败工作领域。强化对权力的监督，加大纠风和专项治理力度，切实解决损害群众利益的突出问题，坚决查处违法违纪案件，以廉政建设和反腐败斗争实效保障发展、取信于民!

各位代表!伴随着改革开放的春风，驻马店走过了一段辉煌历程，而今我们又站到了一个新的历史起点上。美好前景催人奋进，艰辛征程任重道远。加快崛起的驻马店，天时、地利、人和，万事俱备;880多万天中儿女，心齐、气顺、劲足，蓄势待发。让我们在省委、省政府和市委的领导下，在中原经济区建设的伟大征程中，在这片富饶美丽的天中大地上，凝聚全市人民的智慧和力量，以更加振奋的精神、更加开阔的视野、更加务实的作风，不断解放思想、锐意进取，团结拼搏、克难攻坚，努力开创我市经济社会发展的新局面，以优异成绩迎接党的十八大胜利召开!
